# Microglial metabolic reprogramming drives the therapeutic effects of bavachinin on brain network function and memory in Alzheimer’s disease

**DOI:** 10.3389/fphar.2026.1839602

**Published:** 2026-05-28

**Authors:** Yang Zou, Yanni Lin, Chenglong Zhang, Yang Li, Xiaoping Chen, Xingxing Ma, Kehan Chen, Bowei Liang, Peng Liang

**Affiliations:** 1 Center for Medical Research, the First People’s Hospital of Yulin, The Sixth Affiliated Hospital of Guangxi Medical University, Yulin, China; 2 Neurology Department, The First People’s Hospital of Yulin, the Sixth Affiliated Hospital of Guangxi Medical University, Yulin, China; 3 Guangxi Key Laboratory of Special Biomedicine and Advanced Institute for Brain and Intelligence, School of Medicine, Guangxi University, Nanning, China; 4 Department of Pharmacy, the First People’s Hospital of Yulin, The Sixth Affiliated Hospital of Guangxi Medical University, Yulin, China; 5 Center for Respiratory Medicine, the First People’s Hospital of Yulin, The Sixth Affiliated Hospital of Guangxi Medical University, Yulin, China

**Keywords:** alzheimer’s disease, aβ, bavachinin, microglia, network function

## Abstract

**Introduction:**

Alzheimer’s disease (AD) is a progressive neurodegenerative disorder characterized by the accumulation of amyloid-β (Aβ) plaques and pervasive cognitive decline. Bavachinin, a natural flavonoid derived from the traditional medicinal herb Psoralea corylifolia, has previously been demonstrated to inhibit Aβ aggregation *in vitro*. However, its potential to alleviate cognitive impairment, restore large-scale brain network dysfunctions, and mitigate AD-related pathology *in vivo* remains elusive.

**Methods:**

In this study, we systematically evaluated the therapeutic efficacy of bavachinin on AD-associated pathology, cortical slow-wave activity (SWA), and behavioral phenotypes in 5xFAD transgenic mice. We utilized behavioral assessments to evaluate learning and memory, mesoscopic wide-field calcium imaging to assess cortical network dynamics, and histological analyses to measure cerebral Aβ deposition. Furthermore, pharmacological inhibition was employed to investigate the mechanistic role of mitochondrial oxidative phosphorylation (OXPHOS).

**Results:**

Behavioral assessments revealed that bavachinin administration significantly rescued deficits in learning and memory. Mesoscopic wide-field calcium imaging further demonstrated that bavachinin substantially enhanced the synchrony of cortical SWA while reducing its frequency in 5xFAD mice, indicating a restoration of network-level dynamics. Histological analyses confirmed a marked reduction in cerebral Aβ deposition, which occurred independent of Aβ production pathways. Mechanistically, bavachinin bolstered microglial Aβ phagocytosis and chemotactic migration by promoting mitochondrial OXPHOS, thereby revitalizing cellular energy metabolism. Notably, pharmacological inhibition of OXPHOS partially abrogated the therapeutic benefits of bavachinin, suggesting that the augmentation of mitochondrial function is a requisite for its anti-AD effects.

**Discussion:**

In summary, bavachinin alleviates cognitive impairment and neuropathology in AD model mice by driving microglial metabolic reprogramming and facilitating Aβ clearance. These findings highlight its robust potential as a therapeutic candidate for AD.

## 1 Introduction

Alzheimer’s disease (AD) is a slowly progressive neurodegenerative disorder with insidious onset and represents the most common form of dementia among the elderly (Scheltens et al., 2021). Its hallmark pathological features include the accumulation of amyloid-β (Aβ) plaques, tau protein hyperphosphorylation, synaptic dysfunction, and neuronal loss (Scheltens et al., 2021). Extensive research has identified abnormal Aβ accumulation and aggregation in the brain as a key driving factor in AD pathogenesis (Hardy and Selkoe, 2002). Consequently, many recent therapeutic strategies have focused on either reducing Aβ production or enhancing its clearance (Weiner, 2025). Among these, monoclonal antibodies targeting Aβ, such as Donanemab and Lecanemab, have shown efficacy in removing Aβ plaques (Sims et al., 2023; van Dyck et al., 2023). However, their clinical benefits in improving cognitive function remain modest (Lian et al., 2024). This limitation suggests that the success of Aβ clearance relies not only on pharmacological intervention but also on the efficiency of endogenous clearance mechanisms, particularly the functional capacity of microglia, which play a central role in Aβ removal (van Olst et al., 2025).

Microglia are the primary immune cells of the central nervous system and play a pivotal role in clearing Aβ and maintaining homeostasis within the neural microenvironment (Fumagalli et al., 2025). However, as AD progresses, microglia undergo functional decline, characterized by reduced Aβ clearance capacity, impaired migratory behavior, and metabolic dysfunction (Hansen et al., 2018). These impairments contribute to further Aβ accumulation and exacerbate neural network deterioration (Heppner et al., 2015). Growing evidence suggests that restoring microglial function, particularly by enhancing their ability to clear Aβ, may represent a promising therapeutic strategy for alleviating AD pathology (Leng et al., 2022; Yang et al., 2024). Since Aβ clearance is a highly energy-dependent process (Ulland et al., 2017; van Olst et al., 2025), improving the metabolic capacity of microglia, especially by enhancing mitochondrial oxidative phosphorylation (OXPHOS), may be essential for boosting their clearance efficiency and overcoming the limitations of current treatment approaches.

Beyond cellular pathology, accumulating evidence indicates that neural network dysfunction is a direct driver of cognitive impairment in AD (Fultz et al., 2019). Specifically, *in vivo* wide-field calcium imaging has revealed that AD brains exhibit impaired cortical slow-wave activity (SWA), characterized by disrupted propagation of low-frequency oscillations (0.1–3 Hz) (Busche et al., 2015). SWA is physiologically critical, serving as a fundamental mechanism for memory consolidation and the clearance of cerebral metabolic waste (Niknazar et al., 2022; Xie et al., 2013). Importantly, the restoration of physiological SWA patterns has been directly linked to the recovery of cognitive function (Busche et al., 2015; Zou et al., 2025). Therefore, identifying therapeutic agents capable of restoring neural network function, particularly SWA, represents a vital strategy for improving cognitive outcomes in AD.

Bavachinin is a natural flavonoid compound extracted from the traditional Chinese medicinal herb Psoralea corylifolia, known for its diverse biological activities, including antioxidant and anti-tumor effects (Zeng et al., 2025). While previous studies have demonstrated that bavachinin can modulate Aβ aggregation *in vitro* (Chen et al., 2013), its impact on pathology, brain SWA and cognitive function *in vivo* in the context of AD remains poorly understood. In particular, it is unclear whether bavachinin exerts its therapeutic potential by influencing microglial function. To address this gap, the present study systematically investigates whether bavachinin mitigates brain SWA and cognitive deficits in an AD mouse model by enhancing microglia-mediated Aβ clearance.

In this study, we evaluated the therapeutic efficacy and underlying mechanisms of bavachinin using the 5xFAD transgenic mouse model. We demonstrate that a 4-week treatment regimen (10 mg/kg, administered 3 times per week) significantly ameliorates cognitive deficits, including working memory (Y-maze), short-term memory (Novel Object Recognition Test), and long-term spatial memory (Morris Water Maze). At the network level, cortical wide-field calcium imaging revealed that bavachinin restores SWA by enhancing long-range coherence and reducing oscillation frequency. Mechanistically, combined immunostaining and metabolic analyses indicate that bavachinin promotes microglial OXPHOS and ATP production, thereby empowering microglial migration and Aβ clearance. Importantly, pharmacological validation suggests that this enhancement of microglial mitochondrial function serves as the cellular basis for the observed improvements in cortical SWA and cognitive performance. These findings highlight bavachinin as a promising therapeutic candidate for AD.

## 2 Materials and methods

### 2.1 Animals and drug administration

Six-to seven-month-old 5xFAD transgenic mice (Tg6799 line) (Oakley et al., 2006)and age-matched wild-type (WT) littermates of both sexes were used in this study. All animals were housed in a temperature- and humidity-controlled facility under a standard 12-h light/dark cycle, with *ad libitum* access to standard rodent chow and water. All experimental procedures were conducted in strict accordance with the guidelines approved by the Committee for the Use of Experimental Animals at Guangxi University.

**FIGURE 1:**
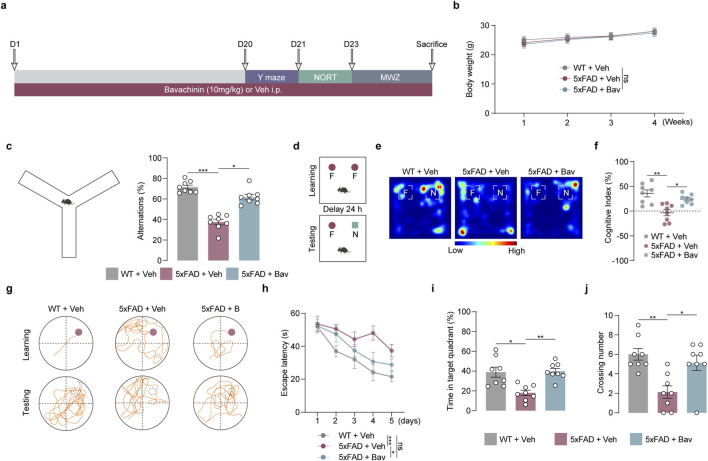
Bavachinin ameliorates learning and memory deficits in 5xFAD mice. **(a)** Experimental workflow schematic. **(b)** Statistical plot shows the weekly changes in body weight (n = 8 mice per group, two-way ANOVA). **(c)** Left: Schematic diagram of the Y-maze spontaneous alternation test. Right: Statistical plot shows the percentage of spontaneous alternations during the 20-min exploration period (n = 8 mice per group, one-way ANOVA). Schematic, **(d)** Schematic diagram of the novel object recognition test (NORT) workflow. **(e)** The trajectory heatmap shows object recognition memory during the testing phase. **(f)** Statistical plot showing the cognitive index (%) during the testing phase of the NORT (n = 8 mice per group, one-way ANOVA). **(g)** Representative swim trajectories during the Morris water maze test. Top: Trajectory on the final day of the acquisition phase, reflecting spatial learning performance. Bottom: Trajectory during the probe trial, indicating memory retention of the platform location. **(h)** Statistical plot shows the daily escape latency during the acquisition phase of the Morris water maze (n = 8 mice per group, two-way ANOVA). **(i)** Statistical plot shows the percentage of time spent in the target quadrant during the probe trial of the Morris water maze (n = 8 mice per group, one-way ANOVA). **(j)** Statistical plot shows the number of platform crossings during the probe trial of the Morris water maze (n = 8 mice per group, one-way ANOVA).Each dot represents an individual animal. Error bars represent mean ± SEM. *P < 0.05, **P < 0.01, ***P < 0.001, ns, no significance.

Bavachinin (CAS number: 19879-30-2; MedChemExpress: HY-N0234; Purity: 99.95%) was dissolved in a vehicle solution comprising 10% DMSO (CAS number: 67-68-5; MedChemExpress: HY-Y0320; Purity: 99.99%), 40% PEG300 (CAS number: 25322-68-3; MedChemExpress: HY-Y0873; Purity: 97.0%), 5% Tween-80 (CAS number: 9005-65-6; MedChemExpress: HY-Y1891), and 45% saline (SOUTHWEST PHARMACEUTICAL). Bavachinin was administered via intraperitoneal (i.p.) injection at a dose of 10 mg/kg, three times per week. This dosage was selected based on previously reported *in vivo* anti-inflammatory efficacy of Bavachinin at comparable concentrations (12.5 mg/kg) in mouse models (Deng et al., 2023), and was further optimized for the long-term treatment requirements of our 5xFAD model to ensure sustained therapeutic effects while mitigating cumulative injection-related stress. Bavachinin (10 mg/kg, i. p.) was administered three times per week for a total duration of 4 weeks, encompassing the entire behavioral assessment phase (Figure 1a).

Additionally, to investigate the necessity of OXPHOS in bavachinin-mediated neuroprotection, the OXPHOS inhibitor oligomycin (CAS number: 1404-19-9; MedChemExpress: HY-N6782; Purity: 98.64%) or vehicle (DMSO) was administered. For intracerebroventricular (i.c.v.) administration, mice were implanted with a guide cannula into the right lateral ventricle. After post-operative recovery, oligomycin or vehicle was administered through the cannula at a volume of 1 μL per injection (Concentration of oligomycin: 1 μg/μL), three times per week for 4 weeks.

### 2.2 Y-maze spontaneous alternation test

Spatial working memory was assessed using the Y-maze spontaneous alternation test (Zhang et al., 2026). The apparatus consisted of three white, opaque arms oriented at 120° angles to each other. For each trial, mice were individually placed at the distal end of one arm and allowed to explore the maze freely for 20 min. Behavior was recorded using a ceiling-mounted video camera. Arm entries were manually scored by an investigator blinded to the experimental conditions. An arm entry was defined as the placement of all four paws into an arm. Spontaneous alternation was defined as a triad of consecutive entries into all three distinct arms (e.g., A-B-C, B-C-A, or C-A-B). The percentage of spontaneous alternation was calculated using the following formula:

$$Alternations\left(\%\right)=\frac{\mathrm{number}\;\mathrm{of}\;\mathrm{spontaneous}\;\mathrm{alternations}}{\mathrm{total}\;\mathrm{number}\;\mathrm{of}\;\mathrm{arm}\;\mathrm{entries}-2}\;*100\%$$

### 2.3 NORT

The Novel Object Recognition Test (NORT) was employed to evaluate recognition memory (Zou et al., 2025). The protocol consisted of a habituation phase, a familiarization phase, and a test phase. Initially, mice were placed in an open-field arena and allowed to explore the empty chamber for 3 min to facilitate habituation. Subsequently, two identical objects were positioned symmetrically within the arena, and the mice were allowed to explore for an additional 10 min (familiarization phase). Following this session, mice were returned to their home cages. After a 24-h retention interval, the test phase was conducted in the same arena. One of the familiar objects was replaced with a novel object, while the other remained in its original position. Mice were reintroduced to the arena and allowed to explore freely for 10 min. Behavior was recorded via a top-mounted video camera, and the time spent exploring each object was analyzed using a custom MATLAB script. The discrimination index (DI, %) was calculated as follows:

$$\mathrm{Cognitive}\;\mathrm{index}\left(\%\right)=\frac{\mathrm{time}\;\mathrm{exploring}\;\mathrm{the}\;\mathrm{novel}\;\mathrm{object‐time}\mathrm{exploring}\;\mathrm{the}\;\mathrm{familiar}\;\mathrm{object}\;}{\left(\mathrm{total}\;\mathrm{exploration}\;\mathrm{time}\;\right)}\;*100\%$$

### 2.4 Morris water maze test

Spatial learning and reference memory were evaluated using the Morris water maze task (Vorhees and Williams, 2006). The apparatus consisted of a circular pool (1.50 m diameter, 0.50 m height) filled with water (22 °C ± 1 °C) rendered opaque by the addition of titanium dioxide (CAS number: 13463-67-7; Zhongyexincai: ZY-TiO_2_-8; Purity: 99.95%). A hidden circular escape platform (10 cm diameter) was submerged 1 cm below the water surface and positioned at the center of a designated target quadrant (Quadrant I). The pool was virtually divided into four equal quadrants (I, II, III, and IV). Animal trajectories were recorded via a ceiling-mounted camera and analyzed using a custom MATLAB-based tracking system. The spatial acquisition phase was conducted over five consecutive days, with four trials per day. For each trial, mice were released into the water facing the pool wall from one of three pseudo-randomly assigned starting points (located in quadrants II, III, or IV). Each trial had a maximum duration of 60 s. If a mouse located the platform within this time, it was allowed to remain on it for 60 s. If the mouse failed to locate the platform within 60 s, it was gently guided to the platform by the experimenter. To ensure memory consolidation, mice were required to stay on the platform for a cumulative duration of 60 s; if a mouse jumped off, it was guided back until the time requirement was met. On day 6, a probe trial was performed to assess memory retention. The platform was removed, and mice were released from a fixed starting point in the opposite quadrant (Quadrant III). Each mouse was allowed to swim freely for 60 s. Spatial memory was quantified by analyzing the time spent in the target quadrant and the number of crossings over the former platform location.

### 2.5 Wide-field Ca^2+^ imaging *in vivo*

The overall experimental procedures were performed in accordance with our previously established protocols (Zou et al., 2025). Surgical reparation mice were anesthetized with an initial induction of 3% isoflurane (RWD), followed by maintenance at 0.8%–1.0% in air. Depth of anesthesia was monitored by the absence of pedal withdrawal reflexes, and respiration was maintained at 80–110 breaths/min. Body temperature was kept constant at 37 °C ± 0.5 °C using a heating pad, and ophthalmic ointment (Bepanthen, Bayer) was applied to prevent corneal desiccation. Following scalp incision and exposure of the skull, a custom-designed recording chamber was secured using dental adhesive and light-curable resin (Tetric N-Flow, Ivoclar Vivadent AG). For comprehensive cortical imaging, a cranial window was created over both hemispheres, with meticulous care taken to preserve the integrity of the dura mater and underlying cortex. In specific experiments involving cannula implantation, the cranial window was restricted to the relevant hemisphere. During the procedure, the recording chamber was continuously perfused with artificial cerebrospinal fluid (ACSF, G-CLONE) containing (in mM): 125 NaCl, 4.5 KCl, 26 NaHCO3, 1.25 NaH2PO4, 2 CaCl2, 1 MgCl2, and 20 glucose (pH 7.4).

Cortical calcium dynamics were visualized using the fluorescent calcium indicator Cal-520 AM (AAT Bioquest). The dye was prepared by dissolving Cal-520 AM (10 mM) in 20% Pluronic F-127/DMSO (Thermo Fisher Scientific) and subsequently diluting it with ACSF to a working concentration of 0.5 mM. Multi-site cortical loading was performed using a glass micropipette coupled to a microinjector (RWD Life Science), delivering 500 nL of the solution per site at a rate of 3 nL/s. This convection-enhanced loading protocol achieved extensive labeling across the cortical surface. Wide-field imaging was performed using a fluorescence microscope equipped with a 2×/0.08 NA apochromatic objective (Olympus). Images were acquired using a high-sensitivity sCMOS camera (Dhyana 400BSI, TUCSEN) controlled by Mosaic v1.4 software. Data were captured at a frame rate of 30 Hz with a spatial resolution of 1,024 × 1,024 pixels.

Offline data analysis was conducted using custom-written scripts in LabVIEW 2014 (LOTOS program), Igor Pro 5.0 (Wavemetrics), and MATLAB 2016b (MathWorks). Regions of interest (ROIs) were defined, and raw fluorescence signals were converted to relative fluorescence changes (Delta F/F) using the formula Delta F/F = (F - F_0)/F_0, where F_0 represents the baseline fluorescence defined as the 25th percentile of the signal intensity for each ROI. To remove physiological noise, specifically heartbeat-induced artifacts (>4 Hz), a Fast Fourier Transform (FFT)-based filter was applied using MATLAB. To evaluate network synchronization, the cortex was segmented into four functional areas per hemisphere (anterior to posterior): frontal, motor, somatosensory, and visual cortices. Spatial coherence was quantified by calculating the correlation of mean calcium signals between these regions over 30-s epochs. For the analysis of calcium transient frequency, a peak detection algorithm was employed. The Delta F/F traces were smoothed (window span = 3), and putative peaks were identified based on a minimum prominence threshold equal to the standard deviation of the signal. The detection logic enforced a refractory period (maximum 1 peak/s) in the initial pass, followed by a secondary refinement based on average peak width to confirm the final transient events.

### 2.6 Thioflavin S staining of amyloid plaques

To visualize amyloid plaques, Thioflavin S staining was performed on brain sections (Zou et al., 2025). Mice were deeply anesthetized with sodium pentobarbital and transcardially perfused with ice-cold physiological saline. Brains were rapidly harvested and sagittally bisected. One hemisphere was snap-frozen in liquid nitrogen for subsequent molecular analyses, while the contralateral hemisphere was fixed and cryoprotected in 4% paraformaldehyde (PFA) containing 20% sucrose at 4 °C for 48 h. Coronal sections (50 µm thick) were obtained using a cryostat (Leica CM 1950). Free-floating sections were incubated in a 0.02% Thioflavin S solution (dissolved in 50% ethanol) for 30 min at room temperature in the dark. Following incubation, sections were differentiated in 50% ethanol, washed extensively with phosphate-buffered saline (PBS), and mounted onto glass slides. Slides were coverslipped with an antifade mounting medium, and images were acquired using a Zeiss LSM880 confocal microscope.

### 2.7 Quantification of Aβ40 and Aβ42 by ELISA

To quantify soluble and insoluble Aβ40 and Aβ42 levels, enzyme-linked immunosorbent assay (ELISA) was performed on brain homogenates (Zou et al., 2025). Mice designated for ELISA and enzymatic activity assays were deeply anesthetized and transcardially perfused with cold phosphate-buffered saline (PBS) to eliminate blood contamination. Brains were quickly harvested and snap-frozen in liquid nitrogen. Frozen brain tissues were pulverized using a pre-chilled mortar and pestle under liquid nitrogen. Homogenization was carried out in Tris-buffered saline (TBS), followed by centrifugation at 13,000 × g for 1 h at 4 °C. The resulting supernatant was collected as the soluble Aβ fraction. The remaining pellet was extracted with SDS-containing buffer and centrifuged again at 13,000 × g for 1 h at 4 °C. The resulting supernatant was collected as the insoluble Aβ fraction. To measure circulating Aβ levels, plasma samples were collected from the left ventricle using EDTA-pre-rinsed Eppendorf tubes. Blood samples were centrifuged at 2000 rpm for 20 min at 4 °C, and the supernatant was collected as plasma. Aβ40 and Aβ42 concentrations in both brain fractions and plasma were measured using commercial ELISA kits (Elabscience) according to the manufacturer’s instructions. Final concentrations were normalized to total protein content.

### 2.8 Quantification of APP, BACE1, and γ-secretase subunit PEN2 in brain homogenates by ELISA

To quantify the expression levels of amyloid precursor protein (APP), β-site APP cleaving enzyme 1 (BACE1), and the γ-secretase subunit presenilin enhancer 2 (PEN2) in mouse brain tissue, ELISA assays were performed using commercial kits (Zou et al., 2025). Mice were deeply anesthetized and transcardially perfused with ice-cold phosphate-buffered saline (PBS) to remove circulating blood. Brains were rapidly extracted and snap-frozen in liquid nitrogen. Frozen brain tissue was homogenized in ice-cold PBS containing protease inhibitor cocktail, followed by centrifugation at 13,000 × g for 15 min at 4 °C. The supernatant was collected and used for ELISA analysis. Quantification of APP, BACE1, and PEN2 was carried out using mouse-specific ELISA kits purchased from EIAab (Wuhan, China): APP (Cat# E0946 m), BACE1 (Cat# E0718 m), and PEN2 (Cat# E3143 m). All assays were conducted according to the manufacturer’s protocols. The measured concentrations were normalized to the total protein content of each sample, determined by BCA protein assay.

### 2.9 Immunofluorescence staining of brain sections

Immunofluorescence staining was performed to assess microglial activation and amyloid deposition in mouse brain tissue (Zou et al., 2025). Mice were deeply anesthetized and transcardially perfused with ice-cold phosphate-buffered saline (PBS), followed by fixation with 4% paraformaldehyde. Brains were post-fixed overnight at 4 °C, cryoprotected in 20% sucrose, and coronally sectioned at a thickness of 50 μm using a cryostat (Leica CM 1950).

For antigen retrieval, brain sections were incubated in 10 mM sodium citrate buffer (Zhongshanjinqiao) at 95 °C for 20 min, then cooled to room temperature. Sections were washed in PBS and permeabilized in PBS containing 0.3% Triton X-100 (Zhongshanjinqiao), followed by blocking with 5% donkey serum for 1 h at room temperature. Sections were then incubated overnight at 4 °C with the following primary antibodies diluted in blocking buffer.

Rabbit anti-Iba1 (1:500; Abcam, catalog no. ab178846)

Rat anti-CD68 (1:500; Abcam, catalog no. ab53444)

Mouse anti-Aβ (clone 6E10, 1:500; BioLegend, catalog no. 803015)

After washing, sections were incubated for 2 h at room temperature with the corresponding secondary antibodies.

Donkey anti-rabbit Alexa Fluor 488 (1:500; Abcam, catalog no. ab150065)

Donkey anti-mouse Alexa Fluor 555 (1:500; Abcam, catalog no. ab150110)

Donkey anti-rat Alexa Fluor 647 (1:500; Abcam, catalog no. ab150155)

Sections were then washed with PBS, mounted on glass slides using antifade mounting medium, and imaged using a Zeiss LSM880 confocal microscope.

### 2.10 BV2 microglial cell culture

The BV2 microglial cell line (SUNNCELL) was used for *in vitro* experiments (Li et al., 2026). Cells were cultured in Dulbecco’s Modified Eagle Medium (SUNNCELL) supplemented with 10% fetal bovine serum and 1% penicillin-streptomycin. Cells were maintained in a humidified incubator at 37 °C with 5% CO_2_.

### 2.11 Cell viability assay (CCK-8)

Cell viability of BV2 microglial cells upon bavachinin treatment was assessed using the CCK8 (Dojindo) assay (Xia et al., 2020). BV2 cells were seeded into 96-well plates at a density of 10,000 cells per well in 100 μL complete DMEM and allowed to adhere overnight. Cells were then treated with increasing concentrations of bavachinin (0, 5, 10, 20, 50, 100, 200, 400, and 800 μM) for 24 h. Following treatment, 10 μL of CCK-8 reagent was added to each well, and the plates were incubated at 37 °C for 1 h. Absorbance was measured at 450 nm using a microplate reader (SpectraMax M2). Cell viability was calculated as a percentage of the absorbance relative to the untreated control group.

### 2.12 Phagocytosis assay using FAM-labeled Aβ_1-42_

To assess microglial phagocytosis of β-amyloid oligomers, a FAM-labeled Aβ_1-42_ peptide (Anaspec) was used (Leng et al., 2022). FAM-Aβ_1-42_ was initially dissolved in DMSO and subjected to ultrasonic treatment for 10 min to ensure uniform dispersion. The solution was then incubated at 37 °C for 24 h to promote oligomerization, followed by incubation at 4 °C for an additional 24 h. After incubation, the solution was centrifuged at 16,000 × g for 15 min at 4 °C, and the resulting supernatant was collected as the oligomeric Aβ_1-42_ (oligo FAM-Aβ_1-42_) preparation.

BV2 microglial cells were seeded into 24-well plates and allowed to adhere. Upon adherence, cells were treated with bavachinin (final concentration: 20 μM) for 24 h. After pretreatment, oligo FAM-Aβ_1-42_ was added to each well at a final concentration of 2 μM and incubated for the indicated time at 37 °C. To inhibit actin-dependent phagocytosis, cytochalasin D (final concentration: 10 μM; CAS number: 22,144–77–0; MedChemExpress: HY-N6682; Purity: 99.0%) was added to designated wells 30 min prior to oligo FAM-Aβ_1-42_ administration as a negative control.

Prior to imaging, cells were washed thoroughly with PBS to remove uninternalized extracellular Aβ. Fluorescent signals were captured using a fluorescence microscope, and images were analyzed for intracellular FAM-Aβ_1-42_ fluorescence intensity as an indicator of phagocytic activity.

### 2.13 Transwell assays

To assess the chemotactic migration of microglial cells, a transwell migration assay was performed using transwell inserts with 8 μm pore polycarbonate membranes (Corning) (Leng et al., 2022). BV2 microglial cells were resuspended in serum-free DMEM and seeded into the upper chamber of the insert at a density of 50,000 cells per well.

The transwell inserts were then placed into 24-well plates containing 500 μL DMEM supplemented with 10% fetal bovine serum (FBS), with or without treatment compounds as indicated. The lower chamber conditions included either 20 μM bavachinin, 2 μM oligomeric Aβ_1-42_, or vehicle control. Cells were incubated at 37 °C with 5% CO_2_ for 24 h.

After incubation, non-migrated cells remaining on the upper side of the membrane were gently removed with a cotton swab. Migrated cells on the lower surface were fixed with 4% paraformaldehyde for 10 min at room temperature, then stained with crystal violet for 10 min.

Stained membranes were imaged under a microscope, and the number of migrated cells was quantified using ImageJ software.

### 2.14 Intracellular ATP measurement

Intracellular ATP levels in BV2 microglial cells were measured using a luminescence-based ATP assay kit (Elabscience, Cat# E-BC-F002), following the manufacturer’s instructions (Feng et al., 2025). BV2 cells were seeded into 12-well plates and pretreated with either bavachinin (final concentration: 20 μM) or oligomeric Aβ_1-42_ (final concentration: 2 μM) for 24 h. After treatment, cells were gently washed with cold phosphate-buffered saline (PBS) to remove residual medium.

Cell lysates were then prepared according to the assay kit protocol, and ATP levels were measured using a SpectraMax M2 microplate reader in chemiluminescence mode. ATP concentrations were calculated based on a standard curve and normalized to the total protein content of each sample, determined by BCA protein assay.

### 2.15 Metabolic flux analysis by seahorse

The extracellular acidification rate (ECAR) and oxygen consumption rate (OCR) of BV2 microglial cells were measured using the Seahorse XFe24 extracellular flux analyzer (Agilent Technologies). The Seahorse XF Glycolysis Stress Test Kit and XF Cell Mito Stress Test Kit (Agilent Technologies) were used to assess glycolytic and mitochondrial function, respectively, following the manufacturer’s instructions (Ge et al., 2024).

BV2 cells were seeded into Seahorse XF24 cell culture microplates at appropriate density. After cell adherence, cells were treated with Bavachinin (final concentration: 20 μM) for 24 h. Subsequently, the assay medium was replaced, and ECAR or OCR measurements were performed according to the respective assay protocols.

Data acquisition and analysis were carried out using the Seahorse XF Wave software (Agilent). All metabolic parameters expressed as milli-pH per minute (mpH/min) for ECAR or picomoles of oxygen consumed per minute (pmol/min) for OCR.

### 2.16 Intracerebroventricular cannulation and drug administration

For intracerebroventricular (i.c.v.) administration of oligomycin, mice were anesthetized with isoflurane (induction at 3%, maintenance at 1.5%) and placed in a stereotaxic apparatus. A stainless-steel guide cannula (RWD Life Science, China) was implanted into the right lateral ventricle using the following coordinates relative to bregma: anteroposterior (AP): 0.0 mm, mediolateral (ML): 1.0 mm, dorsoventral (DV): −2.0 mm (Zou et al., 2025).

The cannula was fixed to the skull using light-curable resin (Ivoclar Vivadent AG) and dental cement. After a post-operative recovery period, oligomycin (1 μg/μL) or vehicle (DMSO) was administered through the cannula at a volume of 1 μL per injection, three times per week.

### 2.17 Statistical analysis

All statistical analyses were performed using GraphPad Prism software (version 9.4.0, GraphPad Software, Inc., Boston, MA, USA). Data are expressed as the mean ± standard error of the mean (SEM). The normality of data distribution was assessed using the Shapiro-Wilk test. For comparisons between two groups, statistical significance was determined using Student’s t-test (unpaired). For comparisons involving three or more groups, one-way analysis of variance (ANOVA) was employed followed by Tukey’s multiple comparisons test. For data involving two independent variables, two-way ANOVA was used, followed by Bonferroni’s *post hoc* test. A p-value of <0.05 was considered statistically significant.

## 3 Results

### 3.1 Bavachinin improves cognitive impairment in 5xFAD mice

The 5xFAD mouse is a widely used transgenic model of AD (Oakley et al., 2006). To determine whether bavachinin treatment can alleviate cognitive deficits in this model, we conducted a series of behavioral tests to assess cognitive function. In this study, 6- to 7-month-old 5xFAD mice (balanced for sex) received intraperitoneal injections of bavachinin (10 mg/kg, i. p. three times per week) or an equal volume of vehicle control for a duration of 4 weeks (Figure 1a). Throughout the treatment period, body weight was regularly monitored, and no significant differences were observed between the bavachinin-treated and vehicle-treated groups (Figure 1b).

Spatial working memory was evaluated using the spontaneous alternation test in a Y-maze (Figure 1c). Compared to age-matched littermate controls, 5xFAD mice exhibited a significantly lower spontaneous alternation rate, indicating substantial impairment in working memory (Figure 1c). Notably, bavachinin-treated 5xFAD mice showed a marked improvement in alternation performance (Figure 1c), suggesting that bavachinin effectively alleviates spatial working memory deficits in this AD mouse model.

Next, we assessed non-spatial memory using the NORT (Figure 1d). During the first phase, mice were placed in an arena with two identical objects and allowed to explore freely for 10 min before being returned to their home cages. In the second phase, 24 h later, the mice were reintroduced to the same arena for another 10-min exploration period, in which one of the original objects was replaced with a novel object while the positions remained unchanged. Cognitive performance was evaluated using the cognitive index. Age-matched littermate control mice showed a clear preference for the novel object, whereas vehicle-treated 5xFAD mice exhibited a significantly reduced preference, indicating impaired object recognition memory (Figures 1e,f). Notably, 5xFAD mice treated with bavachinin demonstrated a partial restoration of novel object preference compared to the vehicle group (Figures 1e,f).

The Morris water maze is widely regarded as the gold standard for assessing spatial learning and memory (Vorhees and Williams, 2006). During the 5-day training period, 5xFAD mice treated with bavachinin displayed significantly shorter escape latencies compared to vehicle-treated 5xFAD mice, with performance approaching that of age-matched littermate controls (Figures 1g,h). In the subsequent probe trial, bavachinin-treated 5xFAD mice spent more time in the target quadrant and crossed the former platform location more frequently (Figures 1i,j), indicating a substantial improvement in spatial learning and memory.

In summary, these experimental results indicate that bavachinin treatment effectively improves cognitive deficits in 5xFAD mice.

### 3.2 Bavachinin restores cortical network coherence and slow-wave activity in 5xFAD mice

**FIGURE 2:**
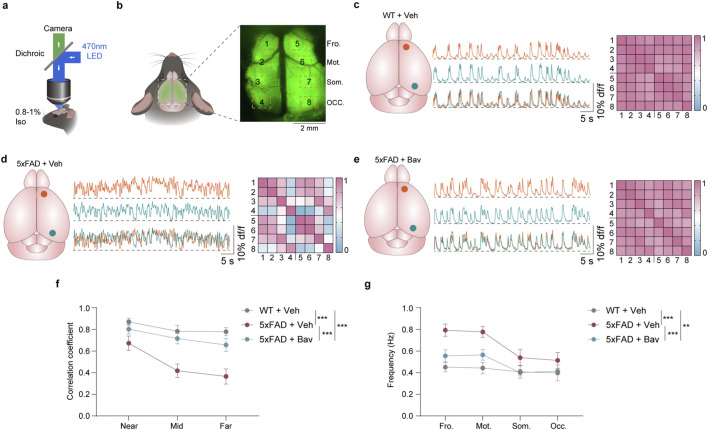
Bavachinin restores cortical SWA and network synchronization in 5xFAD mice. **(a)** Schematic illustration of the *in vivo* wide-field calcium imaging setup using a high-speed sCMOS camera and 470 nm LED illumination in isoflurane-anesthetized mice. **(b)** Representative image of the dorsal cortex showing the expression of the calcium indicator Cal-520 AM. Eight regions of interest (ROIs) were defined across both hemispheres: Frontal (Fro.), Motor (Mot.), Somatosensory (Som.), and Occipital (Occ.) cortices. **(c–e)** Representative relative fluorescence traces (Delta F/F, middle panels) and corresponding correlation matrices (right panels) of spontaneous cortical activity from a **(c)** WT mouse treated with vehicle, **(d)** 5xFAD mouse treated with vehicle, and **(e)** 5xFAD mouse treated with Bavachinin (Bav). The color scale in the matrices represents the correlation coefficient (0–1), indicating the degree of synchronization between ROIs. Note the disrupted rhythmicity and reduced synchronization in the 5xFAD + Veh group, which are rescued in the 5xFAD + Bav group. **(f)** Quantification of correlation coefficients between ROIs separated by varying anatomical distances: Near (adjacent regions), Mid (separated by one region), and Far (separated by two regions). 5xFAD mice show a significant drop in long-range coherence, which is restored by Bavachinin treatment (n = 8 mice per group, two-way ANOVA). **(g)** Quantitative analysis of the oscillation frequency of slow waves across different cortical regions. The aberrant hyperactivity (increased frequency) observed in 5xFAD mice is normalized by Bavachinin treatment (n = 8 mice per group, two-way ANOVA).Error bars represent mean ± SEM. *P < 0.05, **P < 0.01, ***P < 0.001, ns, no significance.

To investigate the impact of bavachinin on cortical network dynamics, we employed wide-field calcium imaging in mice anesthetized with isoflurane (0.8%–1.0%) using a high-speed scientific CMOS camera (Figure 2a). Following a bilateral craniotomy to expose the dorsal cortex (Figure 2b), the calcium indicator Cal-520 AM was bulk-loaded across multiple cortical sites. Under these anesthetic conditions, we observed slow-wave oscillations (<1 Hz) mirroring natural NREM sleep patterns, consistent with prior reports (Busche et al., 2015; Zou et al., 2025). These calcium signals reflect the integrated activity of somatic and neuropil compartments (Busche et al., 2015). To analyze spatiotemporal synchronization, each hemisphere was segmented into four distinct functional areas along the anterior-posterior axis: frontal, motor, somatosensory, and occipital cortices. Inter-regional synchronization was quantified via correlation analysis, where a coefficient approaching 1 indicates high coherence (synchrony) and values near 0 indicate a lack of phase locking.

In vehicle-treated WT mice, calcium imaging revealed highly coherent slow-wave propagation across all ROIs, with signal traces matching closely between regions (Figure 2c). Conversely, age-matched male 5xFAD mice treated with saline exhibited disrupted spatiotemporal patterns; signals within individual regions were disorganized and lacked synchronization with distant areas, indicative of a breakdown in network coherence (Figure 2d). Notably, bavachinin treatment significantly ameliorated this network dysfunction in 5xFAD mice. The treated group displayed restored oscillatory regularity and inter-regional correlation (Figure 2e), exhibiting spatiotemporal patterns resembling those of the healthy WT controls (Figure 2c).

To quantify these observations, we analyzed signal coherence as a function of anatomical distance: “near” (adjacent regions), “mid” (separated by one region), and “far” (separated by two regions, e.g., frontal-occipital). While WT mice maintained high coherence with only minor attenuation over distance, 5xFAD mice showed a precipitous drop in synchronization, particularly between distant cortical areas (Figure 2f). Bavachinin treatment effectively reversed this deficit, maintaining significantly higher coherence across near, mid, and far distances compared to vehicle-treated 5xFAD controls (Figure 2f). Furthermore, the SWA frequency in bavachinin-treated 5xFAD mice was normalized to levels comparable to WT controls, correcting the aberrant frequency increase observed in the vehicle-treated 5xFAD group (Figure 2g). Collectively, these data indicate that a 4-week Bavachinin regimen successfully rescues cortical synchronization and restores physiological SWA properties in the AD mouse model.

### 3.3 Bavachinin significantly reduces aβ burden

**FIGURE 3:**
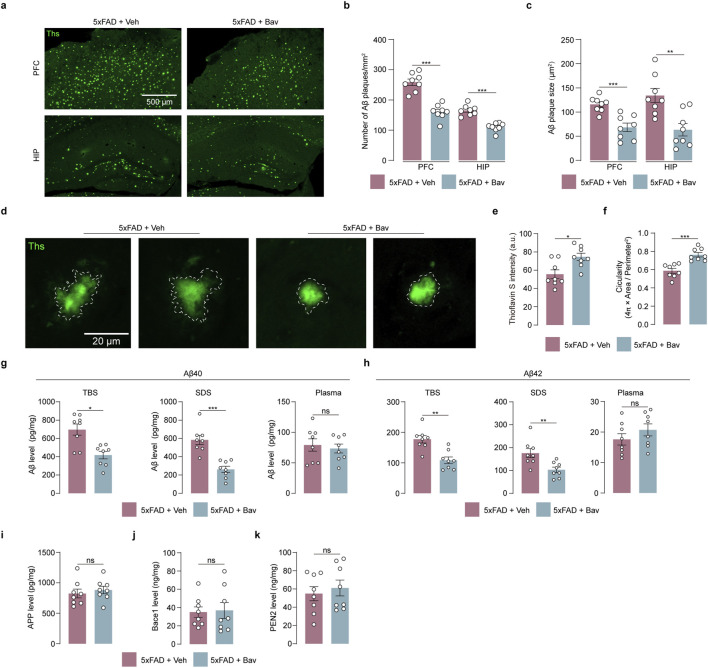
Bavachinin reduces Aβ deposition but not alter Aβ generation. **(a)** Representative images of Aβ plaques in the prefrontal cortex (PFC) and hippocampus (HIP) visualized by thioflavin S staining. **(b)** Statistical plot shows the number of Aβ plaques per square millimeter (n = 8 mice per group, Mann Whitney test). **(c)** Statistical plot shows the differences in Aβ plaque size among experimental groups (n = 8 mice per group, Mann Whitney test). **(d)** Representative image of a single Aβ plaque visualized by Thioflavin S staining. **(e)** Statistical plot shows the fluorescence intensity of individual Aβ plaque among experimental groups (n = 8 mice per group, Mann Whitney test). **(f)** Statistical plot shows the circularity of individual Aβ plaque among experimental groups (n = 8 mice per group, Mann Whitney test). **(g)** Left: Statistical plot shows the level of Aβ40 in TBS-soluble brain homogenates (n = 8 mice per group, Mann Whitney test). Middle: Statistical plot shows the level of Aβ40 in SDS-soluble brain homogenates (n = 8 mice per group, Mann Whitney test). Right: Statistical plot shows the level of Aβ40 in peripheral plasma (n = 8 mice per group, Mann Whitney test). **(h)** Left: Statistical plot shows the level of Aβ42 in TBS-soluble brain homogenates (n = 8 mice per group, Mann Whitney test). Middle: Statistical plot shows the level of Aβ42 in SDS-soluble brain homogenates (n = 8 mice per group, Mann Whitney test). Right: Statistical plot shows the level of Aβ42 in peripheral plasma (n = 8 mice per group, Mann Whitney test). **(i)** Statistical plot showing the expression level of APP in brain homogenate lysates (n = 8 mice per group, Mann Whitney test). **(j)** Statistical plot showing the expression level of Bace1 in brain homogenate lysates (n = 8 mice per group, Mann Whitney test). **(k)** Statistical plot showing the expression level of PEN2 in brain homogenate lysates (n = 8 mice per group, Mann Whitney test).Each dot represents an individual animal. Error bars represent mean ± SEM. *P < 0.05, **P < 0.01, ***P < 0.001, ns, no significance.

Aβ pathology is one of the most prominent hallmarks of AD (Scheltens et al., 2021). Therefore, we investigated whether bavachinin has an impact on Aβ pathology. To assess plaque burden in 5xFAD mice, we performed thioflavin S staining on brain sections and quantitatively analyzed plaque characteristics (Figure 3a). Compared to vehicle-treated 5xFAD mice, those treated with bavachinin exhibited a significant reduction in both the number and size of Aβ plaques in the prefrontal cortex and hippocampus (Figures 3b,c).

We further characterized the morphological architecture of Aβ plaques to evaluate the impact of bavachinin on plaque sequestration (Figure 3d). While plaques in bavachinin-treated mice displayed significantly higher fluorescence intensity and circularity (Figures 3e,f), suggesting enhanced compaction, we sought to provide a more rigorous quantification of this phenomenon using radial intensity profile analysis (Supplementary Figure S1). By delineating the plaque core from its surrounding halo (Supplementary Figures S1a,b), we found that bavachinin treatment resulted in a significantly steeper intensity decay slope compared to the vehicle group (Supplementary Figure S1c), indicating a sharper and more consolidated transition from the fibrillar core to the periphery. Furthermore, the area under the curve (AUC) of the halo region was markedly reduced in bavachinin-treated mice (Supplementary Figure S1d), reflecting a substantial decrease in the accumulation of diffuse Aβ species surrounding the plaque. Together, these multidimensional morphological analyses—encompassing increased intensity, higher circularity, steeper decay slopes, and diminished halo areas—demonstrate that bavachinin treatment promotes the sequestration of diffuse amyloid into dense, compact cores.

To further investigate which components of Aβ are affected by bavachinin, we measured the levels of soluble and insoluble Aβ40 and Aβ42 in whole-brain homogenates using ELISA. Compared to vehicle-treated controls, 5xFAD mice treated with bavachinin showed significantly reduced levels of soluble Aβ40 and Aβ42 in tris-buffered saline (TBS) extracts (Figures 3g,h). Similarly, levels of insoluble Aβ40 and Aβ42 in sodium dodecyl sulfate (SDS) extracts were also markedly decreased in the bavachinin-treated group (Figures 3g h).

To assess whether bavachinin affects peripheral Aβ clearance, we measured Aβ40 and Aβ42 levels in the plasma. No significant differences were observed between bavachinin-treated and vehicle-treated 5xFAD mice (Figures 3g,h), suggesting that the overall reduction in brain Aβ levels induced by bavachinin is not attributable to enhanced BBB-mediated efflux.

We further examined the expression levels of key proteins involved in Aβ production in brain homogenates. Compared to the vehicle group, bavachinin treatment did not significantly alter the expression of APP, BACE1, or PEN2 (Figures 3i–k).

Taken together, these results indicate that bavachinin reduces overall Aβ burden in the brain without affecting Aβ production or its transport across the blood-brain barrier.

### 3.4 Bavachinin enhances microglial phagocytosis of aβ plaques and chemotactic activity

**FIGURE 4:**
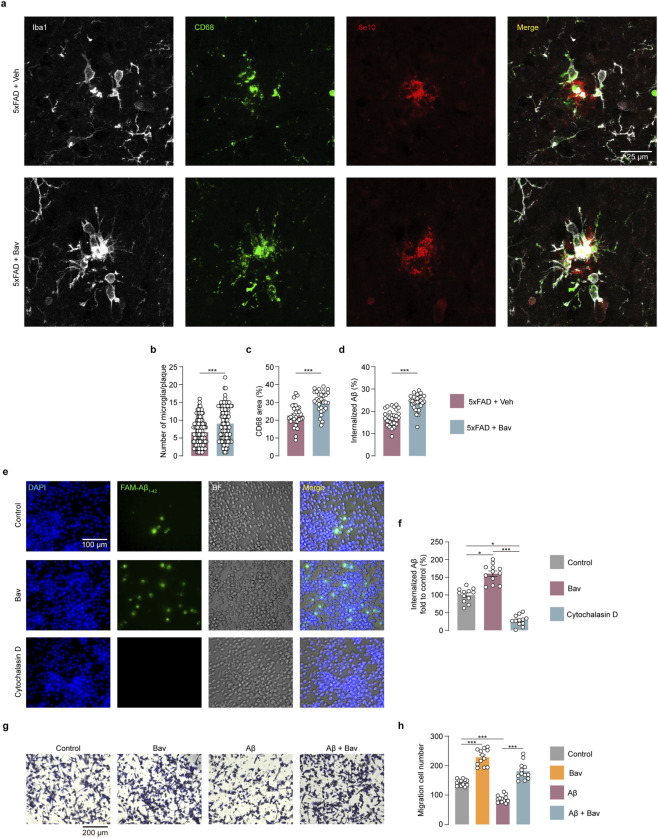
Bavachinin enhances microglial phagocytic and chemotactic activity. **(a)** Representative confocal images showing co-localization of microglia (iba1), Aβ (6e10), and phagosomes (cd68) in the cortex. **(b)** Quantification of plaque-associated microglia. The statistical plot displays the number of Iba1-positive microglia located within a 30 μm radius from the edge of individual Aβ plaques (n = 120 cortical plaques pooled from 8 mice per group, unpaired t-test). **(c)** Analysis of microglial lysosomal capacity. The plot shows the size of CD68-labeled phagosomes within microglia surrounding the plaques, reflecting the lysosomal response to amyloid pathology (n = 30 cortical plaques pooled from 8 mice per group, unpaired t-test). **(d)** Assessment of microglial Aβ internalization. The plot illustrates the percentage of the 6E10-positive area that co-localizes with CD68 signals within the microglial volume. This metric represents the proportion of Aβ successfully engulfed and sequestered into microglial lysosomes (n = 30 cortical plaques pooled from 8 mice per group, unpaired t-test). **(e)** Representative images of *in vitro* Aβ phagocytosis assay. **(f)** Statistical plot shows differences in internalized Aβ levels among treatment groups (Data represent n = 3 independent biological experiments, with 4 technical replicates (wells) per experiment, one-way ANOVA). **(g)** Representative images of the transwell assay. **(h)** Statistical plot shows the number of migrated BV2 microglial cells (Data represent n = 3 independent biological experiments, with 4 technical replicates (wells) per experiment, one-way ANOVA).Error bars represent mean ± SEM. *P < 0.05, **P < 0.01, ***P < 0.001, ns, no significance.

The levels of Aβ in the brain are regulated by its production, degradation, and transport across the BBB (Wang et al., 2017). Given that bavachinin treatment did not affect Aβ production or BBB-mediated transport, we hypothesized that bavachinin may enhance Aβ plaque clearance. Microglia, the resident macrophages of the brain, are primarily responsible for degrading Aβ (Kimura et al., 2025; Wang et al., 2016; Yuan et al., 2016). To investigate this, we performed triple immunofluorescence staining for Iba1 (a microglial marker), CD68 (a lysosomal/phagolysosomal marker), and 6E10 (amyloid plaques) (Figure 4a). Quantitative analysis revealed that bavachinin treatment significantly enhanced microglial recruitment, as evidenced by an increased number of Iba1+ cells clustered within the peri-plaque area (defined as within a 30 μm radius from the plaque perimeter) (Figure 4b). Furthermore, these plaque-associated microglia in the bavachinin group exhibited a marked increase in the average size of CD68^+^ phagolysosomal compartments, suggesting an upregulated lysosomal capacity and a more robust phagocytic response (Figure 4c). To further evaluate the phagocytic efficiency, we quantified the volumetric colocalization of 6E10 and CD68 signals. Bavachinin-treated mice showed a significantly higher percentage of 6E10-positive Aβ internalized within microglial lysosomes (Figure 4d), indicating that bavachinin effectively promotes the microglial engulfment and subsequent degradation of plaque-associated amyloid.

To further examine the effects of bavachinin on microglial phagocytic activity, we performed *in vitro* experiments using BV2 microglial cells (Sabogal-Guáqueta et al., 2023). BV2 cells were treated with various concentrations of bavachinin for 24 h, followed by a CCK-8 assay to assess cell viability. Results showed a concentration-dependent decrease in cell viability at higher doses (50, 100, 200, 400, and 800 μM), prompting the selection of a safer concentration (20 μM) for subsequent experiments (Supplementary Figure S2a). Next, we conducted a phagocytosis assay using FAM-labeled Aβ1-42 (Figure 4e). Consistent with the *in vivo* results, pretreatment with 20 μM bavachinin for 24 h significantly enhanced BV2 phagocytosis of Aβ, while treatment with cytochalasin D (a negative control) markedly suppressed phagocytosis (Figure 4f).

To evaluate the effect of bavachinin on microglial chemotaxis, we performed a transwell migration assay (Figure 4g). Bavachinin pretreatment (20 μM) significantly enhanced the chemotactic ability of BV2 cells, whereas Aβ exposure impaired their chemotactic ability (Figure 4h).

### 3.5 Bavachinin enhances mitochondrial oxidative phosphorylation (OXPHOS) in microglia to support energy supply

**FIGURE 5:**
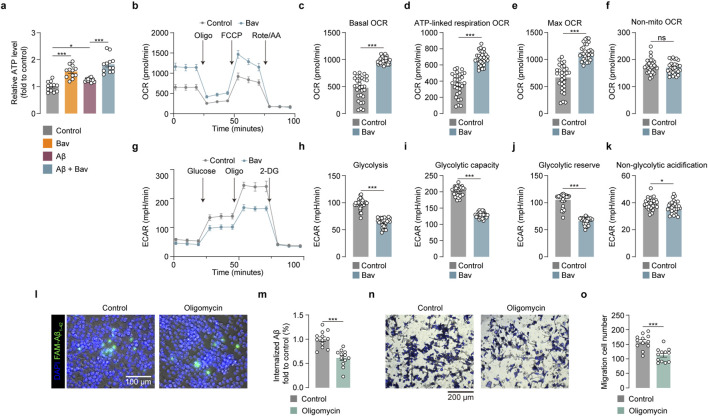
Bavachinin enhances energetic metabolism by promoting mitochondrial OXPHOS in microglia. **(a)** Statistical plot shows differences in ATP levels among different treatment groups (Data represent n = 3 independent biological experiments, with 4 technical replicates (wells) per experiment, one-way ANOVA). **(b)** Measurement of oxygen consumption rate (OCR) using the Seahorse metabolic flux analyzer. **(c–f)** Statistical plots show basal OCR, ATP-linked respiration OCR, maximal OCR, and non-mitochondrial respiration OCR (Data represent n = 3 independent biological experiments, with 10 technical replicates (wells) per experiment, unpaired t-test). **(g)** Measurement of extracellular acidification rate (ECAR) using the Seahorse metabolic flux analyzer. **(h–k)** Statistical plots show basal glycolysis, glycolytic capacity, glycolytic reserve, and non-glycolytic acidification (Data represent n = 3 independent biological experiments, with 10 technical replicates (wells) per experiment, unpaired t-test). **(l)** Representative images of BV2 microglial phagocytosis assay. **(m)** Statistical plot shows the level of Aβ internalized by BV2 microglial cells (Data represent n = 3 independent biological experiments, with 4 technical replicates (wells) per experiment, unpaired t-test). **(n)** Representative images of the BV2 transwell migration assay. **(o)** Statistical plot shows the number of migrated microglial cells (Data represent n = 3 independent biological experiments, with 4 technical replicates (wells) per experiment, unpaired t-test).Error bars represent mean ± SEM. *P < 0.05, **P < 0.01, ***P < 0.001, ns, no significance.

Given that microglial phagocytosis and chemotaxis are highly energy-consuming processes (Chen and Zhong, 2013; Jung et al., 2025), we investigated whether bavachinin treatment affects ATP levels. We found that treatment with bavachinin (20 μM) for 24 h significantly increased ATP levels in BV2 microglial cells, regardless of the presence of Aβ, suggesting that bavachinin alters microglial energy metabolism (Figure 5a).

Glycolysis and mitochondrial OXPHOS are the two major pathways for ATP production (Jung et al., 2025). To determine the metabolic effects of bavachinin on BV2 cells, we performed Seahorse metabolic flux analysis to measure the oxygen consumption rate (OCR, an indicator of OXPHOS) and extracellular acidification rate (ECAR, an indicator of glycolysis) (Figures 5b,g). Quantitative analysis revealed that bavachinin (20 μM, 24 h) significantly increased basal OCR, ATP-linked respiration, and maximal OCR (Figures 5c–e), without affecting non-mitochondrial respiration (Figure 5f). In contrast, bavachinin treatment inhibited glycolytic activity, as evidenced by ECAR measurements (Figure 5h–k).

In summary, these data indicate that bavachinin enhances mitochondrial oxidative phosphorylation in microglia, thereby promoting ATP production to meet the energy demands of microglial function.

To further determine the role of OXPHOS in microglial phagocytic and chemotactic functions, oligomycin (2.5 μM) was used to inhibit OXPHOS in BV2 microglial cells. Results from the Aβ phagocytosis assay showed that oligomycin treatment significantly reduced microglial uptake of Aβ (Figures 5l,m). Similarly, transwell migration assays demonstrated that oligomycin impaired the chemotactic ability of microglia (Figures 5n,o). Taken together, these findings indicate that mitochondrial OXPHOS is essential for supporting microglial phagocytosis and chemotaxis.

### 3.6 Inhibition of mitochondrial OXPHOS attenuates the anti-Alzheimer’s effects of bavachinin

**FIGURE 6:**
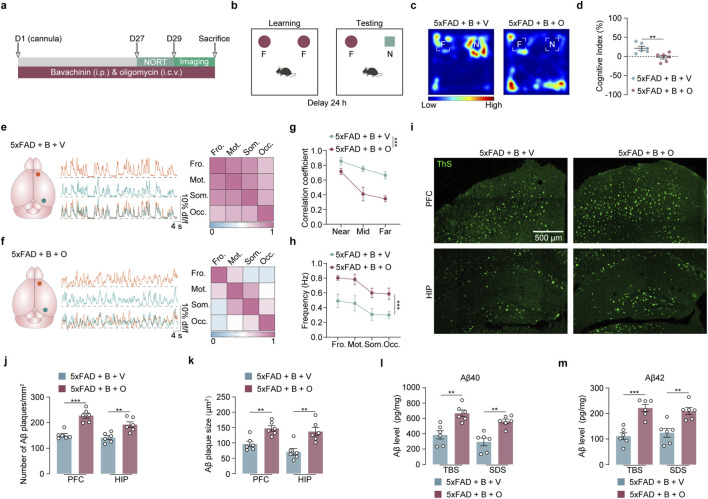
Inhibition of OXPHOS impairs the anti-AD effect of bavachinin. **(a)** Schematic diagram of the *in vivo* drug administration protocol. **(b)** Schematic diagram of the NORT protocol. **(c)** Trajectory heatmaps show object preference for novel versus familiar objects during the test phase across different treatment groups. **(d)** Statistical plot shows the cognitive index across different treatment groups (n = 6 mice per group, Mann Whitney test). **(e,f)** Representative relative fluorescence traces (Delta F/F, middle panels) and corresponding correlation matrices (right panels) of spontaneous cortical activity from a **(e)** 5xFAD mouse treated with B+ V and a **(f)** 5xFAD mouse treated with B+ O. The color scale in the matrices represents the correlation coefficient (0–1), indicating the degree of synchronization between ROIs. **(g)** Quantification of correlation coefficients between ROIs separated by varying anatomical distances: Near (adjacent regions), Mid (separated by one region), and Far (separated by two regions). **(h)** Quantitative analysis of the oscillation frequency of slow waves across different cortical regions. **(i)** Representative images of Aβ plaques labeled with Thioflavin S staining. **(j)** Statistical plot shows the density of Aβ plaques (n = 6 mice per group, Mann Whitney test). **(k)** Statistical plot shows the size of Aβ plaques (n = 6 mice per group, Mann Whitney test). **(l)** Statistical plot shows the level of Aβ40 in brain homogenates (n = 6 mice per group, Mann Whitney test). **(m)** Statistical plot shows the level of Aβ42 in brain homogenates (n = 6 mice per group, Mann Whitney test).

To further determine whether mitochondrial OXPHOS is a key mechanism underlying bavachinin’s ability to alleviate Aβ burden, restore cortical SWA, and improve cognition, we conducted *in vivo* validation experiments (Figure 6a). In 5xFAD mice, oligomycin (an OXPHOS inhibitor, 1 μL per injection, three times per week) or an equal volume of vehicle was administered via intracerebroventricular (i.c.v.) cannulation for 4 consecutive weeks concurrently with bavachinin treatment.

To ensure the cellular preferential and safety of this intervention, we first characterized the morphological responses of various brain cell types to the administered dose of oligomycin in the prefrontal cortex (Supplementary Figure S3). While oligomycin treatment significantly altered microglial architecture—evidenced by reduced soma area, elongated processes, and increased branching complexity (Supplementary Figure S3a–d)—it had no detectable impact on astrocytic morphology or neuronal density in the prefrontal cortex (Supplementary Figure S3e–j). These results suggest that the chosen dosing regimen preferentially compromises the metabolic state of microglia in the Aβ-burdened environment without inducing overt neurotoxicity or astrocytic disruption, providing a reliable experimental window to dissect the role of microglial OXPHOS in the observed rescue effects.

Behavioral assessment using the NORT revealed that the cognitive benefits conferred by bavachinin were abolished by OXPHOS inhibition. As shown in the heatmaps and cognitive index, mice co-treated with oligomycin failed to discriminate the novel object, performing significantly worse than the bavachinin-only group (Figures 6b–d).

Crucially, wide-field calcium imaging demonstrated that the restoration of cortical network dynamics by bavachinin relies on functional OXPHOS. While the 5xFAD + B+ V group exhibited high spatiotemporal coherence and normalized slow-wave oscillation frequency, co-administration of oligomycin disrupted these patterns, leading to reduced inter-regional correlation and aberrant high-frequency oscillations (Figures 6e–h).

Consistently, histological analysis showed that OXPHOS inhibition attenuated bavachinin’s effect on amyloid pathology, resulting in a significantly higher number and size of Aβ plaques in both the prefrontal cortex and hippocampus (Figures 6i–k). ELISA results further confirmed that oligomycin reversed the bavachinin-induced reduction of Aβ40 and Aβ42 levels in both TBS-soluble and SDS-insoluble brain fractions (Figures 6l,m).

Together, these findings suggest that mitochondrial OXPHOS is essential for the therapeutic effects of bavachinin in rescuing cortical network synchronization, reducing Aβ pathology, and improving cognitive function in AD model mice (as summarized in the working model, Supplementary Figure S4).

## 4 Discussion

This study systematically evaluated the therapeutic potential of bavachinin, a brain-penetrant natural flavonoid, in a mouse model of AD, and demonstrated its significant effects in improving cognitive function, restoring cortical network dynamics, and reducing pathological burden. Long-term administration of bavachinin marked improved spatial working memory, object recognition memory, and spatial learning in 5xFAD mice. Crucially, wide-field calcium imaging revealed that bavachinin rescued the disruption of cortical SWA, reestablishing long-range network synchronization. These functional improvements were accompanied by a significant reduction in both the number and size of Aβ plaques. Mechanistically, bavachinin activated mitochondrial OXPHOS, boosting microglial energy metabolism to support enhanced phagocytic and chemotactic activity toward Aβ plaques. Pharmacological validation further indicated that this metabolic reprogramming is essential for the observed recovery of cortical SWA and cognitive performance. Collectively, this study is the first to reveal that bavachinin improves Aβ clearance and restores neural network homeostasis by modulating microglial metabolic states, providing both mechanistic insight and experimental evidence for the development of microglia-targeted therapeutic strategies for AD.

The Aβ cascade hypothesis posits that abnormal production, accumulation, and impaired clearance of Aβ serve as the initiating events in the pathogenesis of AD, leading downstream to tau hyperphosphorylation, synaptic dysfunction, neuroinflammation, and ultimately neuronal loss (Butterfield and Halliwell, 2019; Guo et al., 2020; Hardy and Selkoe, 2002; Wang et al., 2020). Although this hypothesis has faced increasing scrutiny in clinical settings, the central role of Aβ in AD pathophysiology remains widely accepted (Zhang et al., 2023). Numerous clinical and preclinical studies have demonstrated a strong correlation between reduced cerebral Aβ burden and preserved cognitive function (Insel et al., 2016; Roostaei et al., 2017; You et al., 2019). Enhancing Aβ clearance is therefore considered a critical strategy for slowing or halting AD progression, particularly in late-onset AD, where impaired clearance is thought to play a more dominant role than increased production (Krstic and Knuesel, 2013; Mawuenyega et al., 2010; Nalivaeva and Turner, 2019; Valdez-Gaxiola et al., 2024). In this study, we found that bavachinin did not alter the expression of amyloid precursor protein (APP) or its processing enzymes (such as BACE1 and PEN2), nor did it affect plasma Aβ levels. These findings suggest that the reduction in Aβ burden observed with bavachinin treatment is not due to decreased production or enhanced peripheral efflux, but rather the result of improved central clearance. This supports the therapeutic relevance of targeting Aβ clearance mechanisms—particularly through activation of endogenous immune cells—and provides additional evidence in support of the Aβ cascade hypothesis.

Microglia rely on highly dynamic cytoskeletal remodeling and membrane reorganization to carry out their phagocytic and chemotactic functions—processes that demand a stable and sufficient energy supply (Baik et al., 2019; Li et al., 2022; Sangineto et al., 2023; Zhong et al., 2024). Insufficient energy directly impairs microglial ability to sense, migrate toward, and engulf Aβ (Preeti et al., 2022). ATP, the primary energy currency of the cell, is mainly produced through glucose metabolism (de la Monte and Tong, 2014). Upon entering the cell, glucose is first broken down into pyruvate via cytoplasmic glycolysis, yielding 2 molecules of ATP per glucose molecule (Cunnane et al., 2011). Under aerobic conditions, pyruvate enters the mitochondria and undergoes the tricarboxylic acid (TCA) cycle followed by the electron transport chain (ETC.), collectively known as OXPHOS, which generates an additional 26 to 30 molecules of ATP (Leng et al., 2022). In contrast, under anaerobic conditions, pyruvate is reduced to lactate, producing only 2 molecules of ATP in total—substantially less than OXPHOS. In this study, we found that bavachinin significantly increased ATP levels in microglia. Seahorse metabolic flux analysis further confirmed that this effect was driven by enhanced mitochondrial OXPHOS rather than glycolysis. This metabolic reprogramming enables microglia to meet the high energy demands required to maintain effective Aβ phagocytosis and chemotaxis under AD-related pathological conditions. Conversely, when OXPHOS was inhibited with oligomycin, microglial function was compromised, and the beneficial effects of bavachinin on cognition and Aβ clearance were markedly attenuated. These findings highlight the essential role of mitochondrial metabolism in the anti-AD effects of bavachinin and underscore the broader link between immunometabolism and AD pathology, suggesting that targeting microglial energy metabolism represents a promising therapeutic strategy.

What underlying mechanisms link the reduction of Aβ burden to the restoration of cortical SWA? We propose that bavachinin restores network-wide synchrony by simultaneously mitigating Aβ-induced synaptic toxicity. Accumulating evidence suggests that soluble Aβ oligomers are primary drivers of network dysfunction in early AD (Busche et al., 2012). These toxic species are known to impair synaptic glutamate reuptake, leading to pathological glutamate accumulation in the synaptic cleft and subsequent localized neuronal hyperactivity (Zott et al., 2019). This resultant E/I disequilibrium acts as a critical driver for the fragmentation of long-range cortical synchronization, effectively decoupling distant brain regions (Palop and Mucke, 2016; Zott et al., 2024). Furthermore, structural pathologies, such as dystrophic neurites associated with amyloid plaques, may physically impede the propagation of action potentials along axons, further severing the functional connectivity required for coherent oscillations (Yuan et al., 2022). In the present study, bavachinin treatment significantly reduced the levels of soluble Aβ (Figure 6) and mitigated plaque pathology. We propose that by empowering microglia to clear these toxic Aβ species, bavachinin alleviates the synaptic toxicity that drives neuronal hyperactivity. This clearance effectively removes the “noise” in the neural circuit, allowing physiological SWA patterns to re-emerge. This hypothesis is supported by our observation that the recovery of cortical coherence coincides with the reduction in soluble Aβ load, highlighting the restoration of network homeostasis as a key functional outcome of bavachinin-mediated Aβ clearance.

As a natural flavonoid compound derived from the traditional chinese medicinal herb *Psoralea corylifolia*, bavachinin demonstrated promising therapeutic potential against AD in this study. By modulating the metabolic state of microglia, bavachinin enhanced Aβ clearance, thereby reducing Aβ burden and improving cognitive performance. This mechanism of action is distinct from that of conventional Aβ-targeting antibodies or enzyme inhibitors, offering a unique therapeutic advantage. Moreover, throughout the 4-week systemic administration period, no significant difference in body weight was observed between the bavachinin-treated and control groups, suggesting good tolerability and potential safety with long-term treatment. These findings highlight the potential of bavachinin as a candidate therapeutic agent for AD and point to a novel direction for future drug development focused on microglial metabolic modulation.

A notable limitation of the present study is our primary reliance on pharmacological interventions, which inherently lack the absolute cell-type specificity provided by genetic manipulations. For instance, although intraventricular administration of oligomycin is a global metabolic inhibitor, we observed that it preferentially compromised microglial morphology and function at the administered dose, while leaving astrocytic and neuronal structural integrity largely intact (Supplementary Figure S3). This differential sensitivity likely arises from the intense metabolic reprogramming and pre-existing stress of microglia in the Aβ-burdened environment, whereas neurons remain relatively stable and astrocytes can utilize their glycolytic flexibility for energy compensation (Bolaños and Magistretti, 2025). Nevertheless, we cannot entirely rule out the possibility that both bavachinin and oligomycin exert subtle, direct effects on neuronal excitability or astrocytic supportive functions through similar mitochondrial pathways. Due to the current absence of microglia-specific conditional knockout models for key oxidative phosphorylation genes in our experimental setup, we are unable to definitively claim that the observed effects are mediated exclusively via microglia. Future studies utilizing cell-type-specific genetic tools and chemical proteomics will be essential to pinpoint the precise molecular targets of bavachinin and further dissect the distinct metabolic contributions of individual cell populations to plaque compaction and cortical network dynamics.

Another limitation of the current study is the reliance on isoflurane anesthesia during wide-field calcium imaging. Although isoflurane is a GABA_A agonist and NMDA antagonist that alters the brain’s E/I balance, low-dose isoflurane is a standard and validated model for evoking slow-wave oscillations that closely resemble the SWA observed during natural NREM sleep (Busche et al., 2015). Our findings demonstrate that bavachinin can rescue the breakdown of long-range coherence within this specific pharmacological framework. However, whether this restored synchrony persists during natural sleep-wake cycles—and how it directly impacts sleep-dependent memory consolidation—remains to be fully elucidated. Future studies employing long-term EEG recordings in freely moving mice will be essential to confirm the translational potential of bavachinin-mediated SWA restoration for clinical cognitive improvement.

## 5 Conclusion

In summary, our study demonstrates that bavachinin effectively alleviates cognitive impairment and brain network function in the 5xFAD mouse model of AD by enhancing microglia-mediated Aβ clearance. We show that bavachinin promotes microglial phagocytic and migratory activity by boosting mitochondrial OXPHOS, thereby satisfying the high energy demands required for efficient Aβ removal. Crucially, this reduction in amyloid burden translates into the functional restoration of the neural network, characterized by the rescue of cortical SWA and the reestablishment of long-range synchronization. Importantly, the inhibition of oxidative metabolism partially reversed these therapeutic benefits, highlighting the pivotal role of microglial metabolic reprogramming in mediating bavachinin’s ability to restore network homeostasis and combat AD. These findings provide novel insights into the immunometabolic regulation of microglial function and neural network dynamics, suggesting that bavachinin holds promise as a potential therapeutic candidate for AD.

## Data Availability

The original contributions presented in the study are included in the article/Supplementary Material, further inquiries can be directed to the corresponding authors.
