# Supplementary material for: Microglial metabolic reprogramming drives the therapeutic effects of bavachinin on brain network function and memory in Alzheimer’s disease
Source: Front Pharmacol. 2026 May 28;17:1839602. doi: 10.3389/fphar.2026.1839602 (PMC13254562; doi:10.3389/fphar.2026.1839602)
Supplement: Supplementary file 1 [file Supplementaryfile1.docx]

Supplementary Material

# Supplementary Data

Supplementary Material should be uploaded separately on submission. Please include any supplementary data, figures and/or tables.

Supplementary material is not typeset so please ensure that all information is clearly presented, the appropriate caption is included in the file and not in the manuscript, and that the style conforms to the rest of the article.

# Supplementary Figures and Tables

For more information on Supplementary Material and for details on the different file types accepted, please see [here](https://www.frontiersin.org/guidelines/author-guidelines#supplementary-material).

## Supplementary Figures


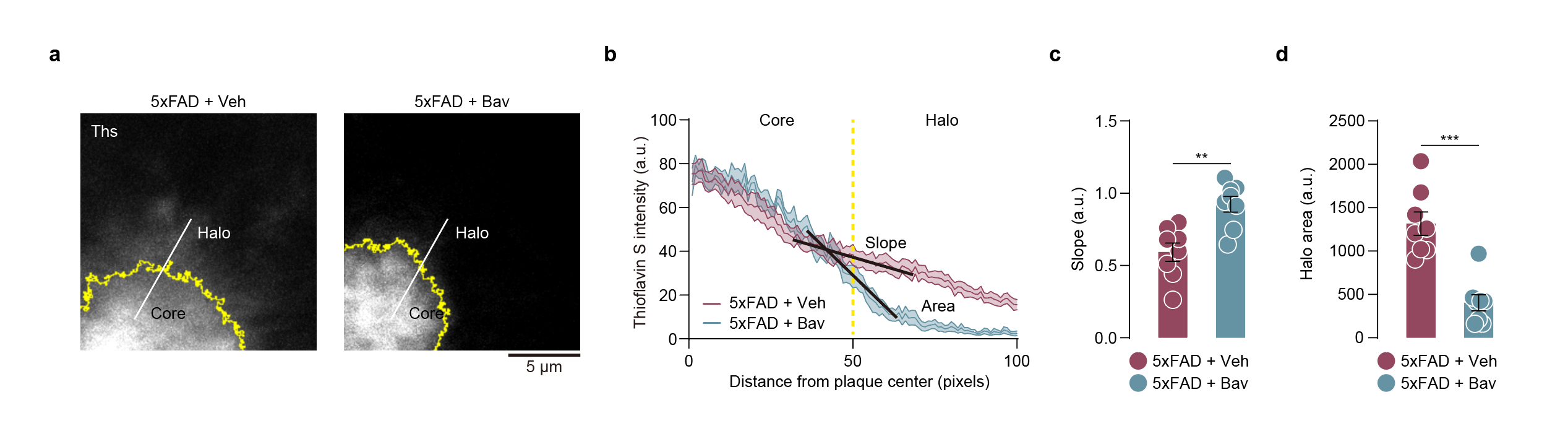


**Supplementary Figure 1.** Quantitative characterization of plaque compaction and core-halo architecture in 5xFAD mice (Relative to Fig. 3).

a. Representative images of Ths-stained amyloid plaques in the cortex of 5xFAD mice treated with Vehicle or bavachinin. Yellow outlines indicate the thresholded boundaries used to distinguish the plaque core from the surrounding halo region.

b. Schematic of the radial fluorescence intensity profile analysis. Ths fluorescence intensity was quantified as a function of distance from the plaque center toward the periphery. The slope of the decay curve represents the rate of fluorescence change, reflecting the sharpness and compactness of the fibrillar core boundary. The area under the curve (AUC) of the distal 50% of the profile represents the integrated Ths fluorescence within the plaque halo region.

c. Quantitative comparison of the decay slope between Veh- and Bav-treated mice. A steeper slope in the Bav group indicates a more abrupt and compact transition from the core to the periphery (n = 8 mice per group, Mann Whitney test).

d. Quantitative analysis of the halo area (AUC), showing a significant reduction in diffuse Ths-positive signals in Bav-treated mice (n = 8 mice per group, Mann Whitney test).

Each dot represents an animal. Error bars represent mean ± SEM. *P < 0.05, **P < 0.01, ***P < 0.001, ns = no significance.


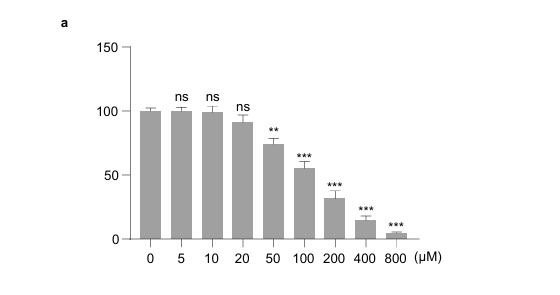


**Supplementary Figure 2.** The viability of BV2 microglial cells under different concentrations of bavachinin.

a. Statistical plot shows the viability of BV2 microglial cells treated with different concentrations of bavachinin, as assessed by the CCK-8 assay (n = 8 technical replicates (wells) per group, one-way ANOVA).

Error bars represent mean ± SEM. *P < 0.05, **P < 0.01, ***P < 0.001, ns = no significance.


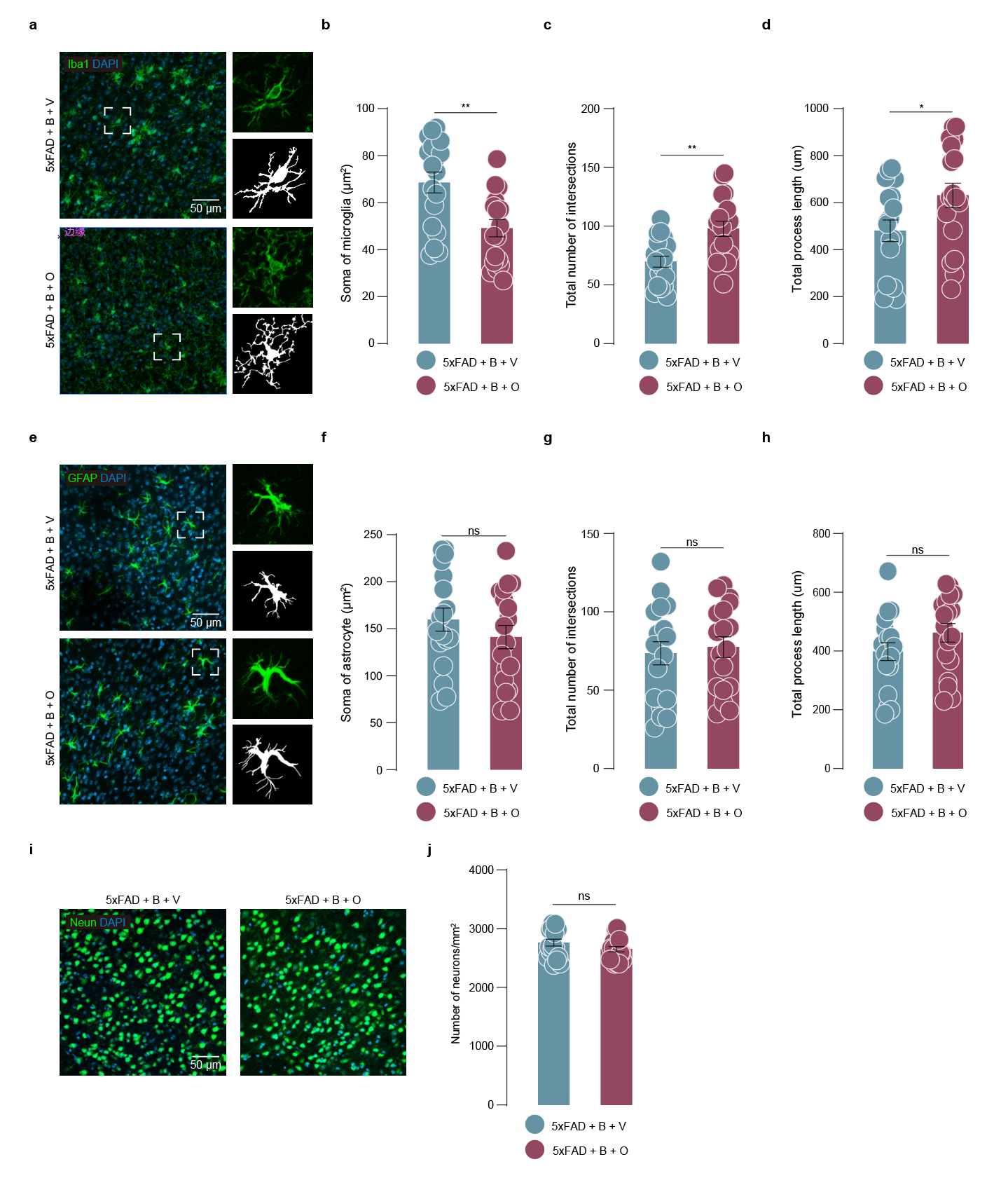


**Supplementary Figure 3.** Differential morphological responses of microglia, astrocytes, and neurons to OXPHOS inhibition in the 5xFAD prefrontal cortex (Relative to Fig. 6).

a. Representative images of microglia. Immunofluorescence staining of Iba1-labeled microglia in the prefrontal cortex of 5xFAD + B + V and 5xFAD + B + O mice.

b. Statistical quantification showing a significant reduction in the soma size of microglia in the B+O group compared to the B+V group (n = 18 microglia pooled from 6 mice per group, unpaired t-test).

c. Quantification of the total number of intersections via Sholl analysis, showing an increase in microglial branching complexity in the 5xFAD + B+ O group, potentially reflecting a dystrophic or

hyper-ramified state under metabolic stress (n = 18 microglia pooled from 6 mice per group, unpaired t-test).

d. Statistical plot showing a significant increase in the total length of microglial processes following oligomycin treatment (n = 18 microglia pooled from 6 mice per group, unpaired t-test).

e. Representative images of astrocytes. Immunofluorescence staining of GFAP-labeled astrocytes in the PFC of 5xFAD + B + V and 5xFAD + B + O mice.

f. Statistical quantification showing no significant difference in the soma size of astrocytes between the 5xFAD + B + V and 5xFAD + B + O groups (n = 18 astrocytes pooled from 6 mice per group, unpaired t-test).

g. Quantification of the total number of intersections via Sholl analysis, showing that astrocytic branching complexity remains unaffected by the current dose of oligomycin (n = 18 astrocytes pooled from 6 mice per group, unpaired t-test).

h. Statistical plot showing that the total process length of astrocytes is preserved in the 5xFAD + B + O group (n = 18 astrocytes pooled from 6 mice per group, unpaired t-test).

i. Representative images of neurons. Immunofluorescence staining of NeuN-labeled neurons in the PFC of 5xFAD + B + V and 5xFAD + B + O mice.

j. Statistical quantification showing that the total number of NeuN-positive neurons remains stable across groups, indicating an absence of acute neurotoxicity at the administered dose (n = 18 fields of view pooled from 6 mice per group, unpaired t-test).


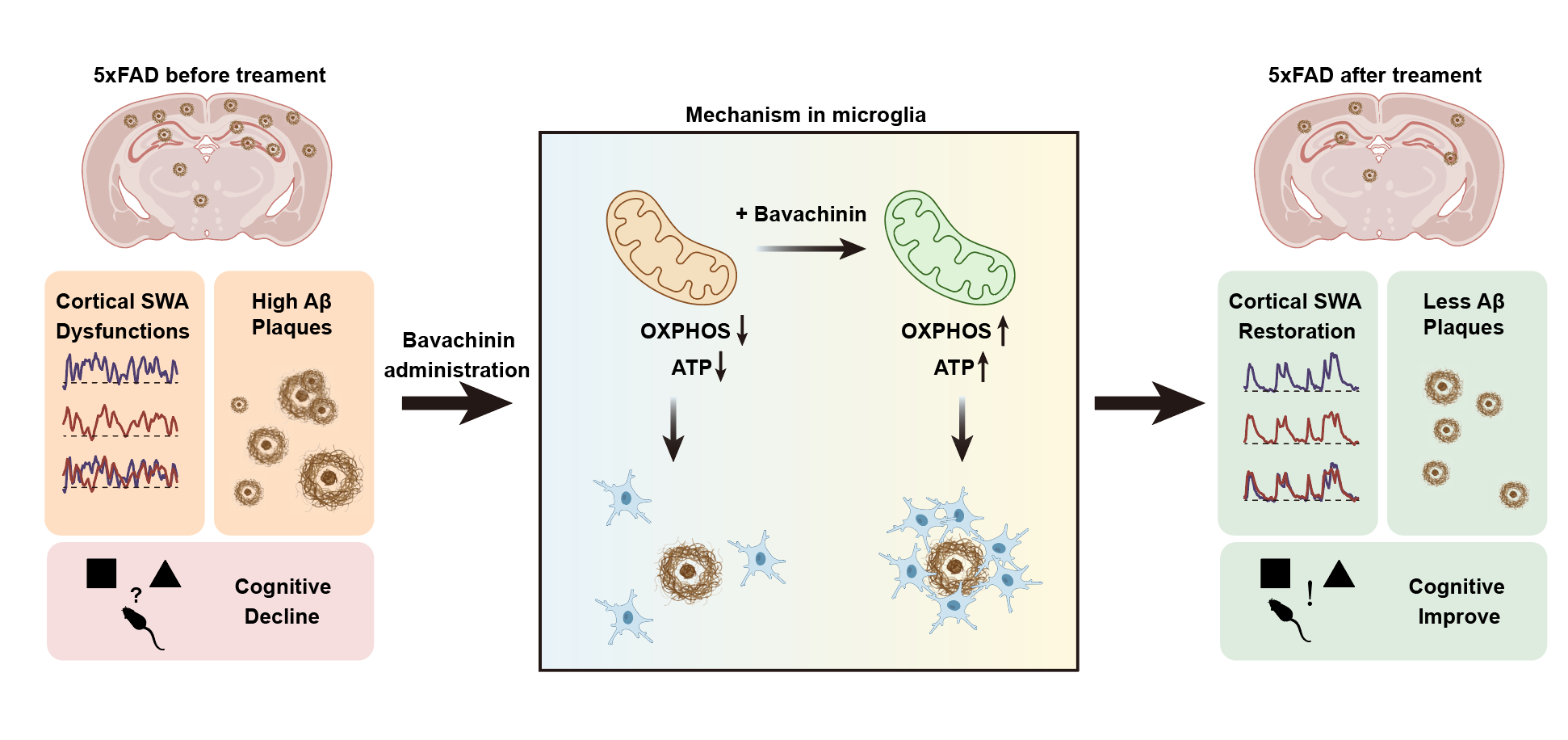


**Supplementary Figure 4**. Bavachinin alleviates Aβ pathology and restores slow-wave activity by boosting microglial OXPHOS-dependent phagocytosis.
